# Cerebral Cryptococcosis Associated with CD4+ T-lymphocytopenia in Non-HIV Patients after SARS-CoV-2 Infection: Case Series in a Specialized Institute in Lima, Peru

**DOI:** 10.3390/tropicalmed8030182

**Published:** 2023-03-22

**Authors:** Juana M. Huamani-Córdova, Miguel Hueda-Zavaleta, Victor Vargas-Bellina, Lourdes Simbron-Ribbeck, Katty del Rosario Chong-Chinchay, Juan Carlos Gómez de la Torre, Vicente A. Benítes-Zapata

**Affiliations:** 1 Instituto Nacional de Ciencias Neurológicas, Lima 15003, Peru; 2 Facultad de Ciencias de la Salud, Universidad Privada de Tacna, Tacna 23003, Peru; mighueda@virtual.upt.pe; 3 Hospital III Daniel Alcides Carrión—Essalud, Tacna 23000, Peru; 4 Roe Clinical Laboratory, Lima 15076, Peru; 5 Unidad de Investigación para la Generación y Síntesis de Evidencias en Salud, Universidad San Ignacio de Loyola, Lima 15024, Peru

**Keywords:** COVID-19, SARS-CoV-2, cryptococcus, cryptococcosis, meningitis, opportunistic infection, T-lymphocytes, central nervous system

## Abstract

Cases of cryptococcosis have been reported in patients with COVID-19. The majority are in patients with severe symptoms or who received immunosuppressants. However, there is still no clear association between COVID-19 and cryptococcosis. We report eight cases of cerebral cryptococcosis associated with CD4+ T lymphocytopenia in non-HIV patients after SARS-CoV-2 infection. The median age was 57 years and 5/8 were male. In addition, 2/8 of patients had diabetes, and 8/8 had a history of mild COVID-19, with a median of 75 days before diagnosis of cerebral cryptococcosis. All patients denied having received prior immunosuppressive therapy. The most frequent symptoms were confusion (8/8), headache (7/8), vomiting (6/8), and nausea (6/8) All patients were diagnosed by isolating Cryptococcus in cerebrospinal fluid. The median CD4+ and CD8+ T lymphocytes were 247 and 173.5, respectively. Other causes of immunosuppression, such as HIV or HTLV infection, were excluded in all patients. Finally, three patients died, and one presented long-term visual and auditory sequelae. The CD4+/CD8+ T lymphocyte count normalized during follow-up in those patients who survived. We hypothesize that CD4+ T lymphocytopenia in the patients in this case series could increase the risk of cryptococcosis after SARS-CoV-2 infection.

## 1. Introduction

More than three years have passed since the emergence of Coronavirus disease 2019 (COVID-19) caused by the severe acute respiratory syndrome coronavirus 2 (SARS-CoV-2) in the city of Wuhan at the end of December 2019. Since then, COVID-19 has spread rapidly worldwide, still threatening global health [1]. Likewise, multiple fungal co-infections such as Aspergillus, Candida, and Mucormycosis have been observed, mainly in patients with critical COVID-19 who required invasive mechanical ventilation or with a lengthy hospital stay [2,3,4]. Patients with severe COVID-19 may present with a pro-inflammatory state with the release of cytokines such as Interleukin (IL)-1, IL-2, IL-6, and tumor necrosis factor (TNF). It has also been described that they may present lymphocytopenia and reduced levels of T cells in peripheral blood, which is a factor associated with a greater risk of severity and mortality from COVID-19 [5]. CD4+ T-cell lymphocytopenia has been reported in some patients with COVID-19, mainly in those with severe symptoms [6,7]; however, its duration is unknown, and it could be a risk factor for opportunistic infections.

Cerebral cryptococcosis is an opportunistic infection that mainly affects patients with Human Immunodeficiency Virus (HIV) infection with CD4+ T-lymphocyte counts lower than 100 cells/µL, and also in transplant recipients, hematological malignancies, idiopathic CD4 lymphocytopenia, and users of prolonged corticosteroid therapy or other immunosuppressants [8]. Its presentation in immunocompetent hosts is infrequent. Although some cases associated with COVID-19 have been reported, these patients generally suffered severe disease, received corticosteroids, and were admitted to the intensive care unit (ICU) [9,10]. We report eight cases of cerebral cryptococcosis in patients without HIV infection, treated at the National Institute of Neurological Sciences (INCN) in Lima, Peru. All patients were diagnosed with COVID-19 by clinical picture and detection of IgM/IgG serum antibodies against COVID-19 and/or polymerase chain reaction (PCR) for SARS-CoV-2 from nasopharyngeal swabs. We included all patients diagnosed with cerebral cryptococcosis by isolating *Cryptococcus*. in cerebrospinal fluid culture and diagnosis or antecedent of confirmed COVID-19 (by RT-PCR of SARS-CoV-2 in those patients with suspicion of COVID-19 with a disease time of less than 7–10 days, or by detecting serum IgM/IgG antibodies against SARS-CoV-2 when disease time was greater than 7–10 days). The INCN Research Ethics Committee approved the study and all survivors gave informed consent.

## 2. Results

### 2.1. Demographic Characteristics

The median age was 57 years, and 5/8 were male. The most frequent comorbidity was diabetes, in 2/8. One patient (case 1) was diagnosed with type II naked lymphocyte syndrome after discharge, and 5/8 had no comorbidity, 8/8 of patients had a history of mild COVID-19, with a median time of 75 days (IQR: 30–180) before diagnosis of cerebral cryptococcosis. Finally, 7/8 had positive serum antibodies against SARS-CoV-2 and 2/8 were RT-PCR SARS-CoV-2 positive at the time of diagnosis of cryptococcosis (Table 1).

### 2.2. Detailed Case Descriptions 

**Figure 1:**
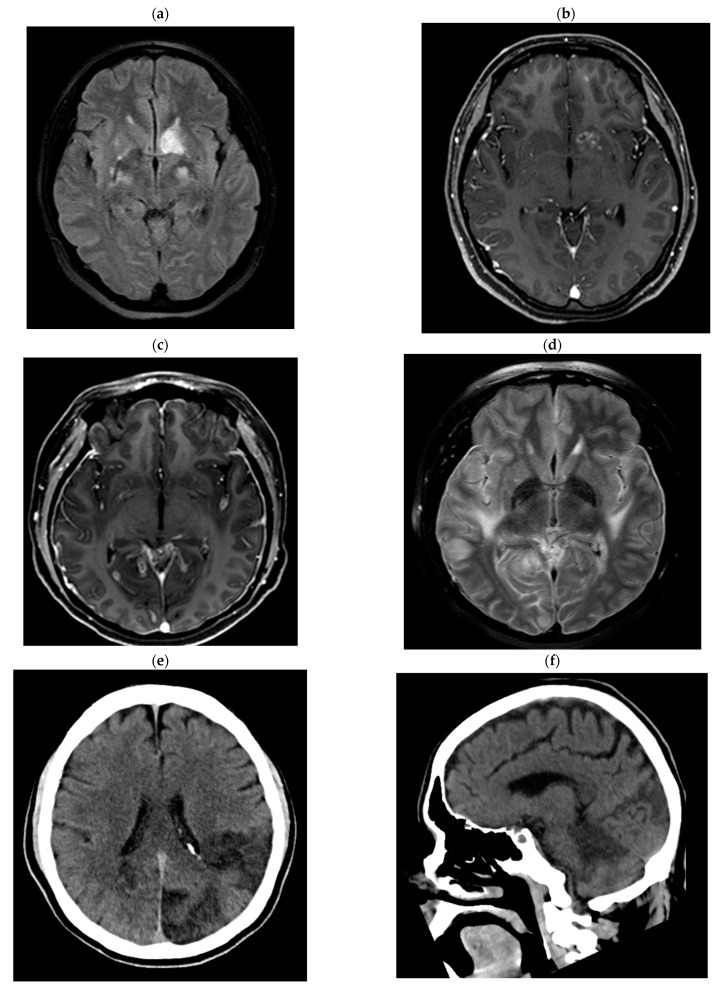

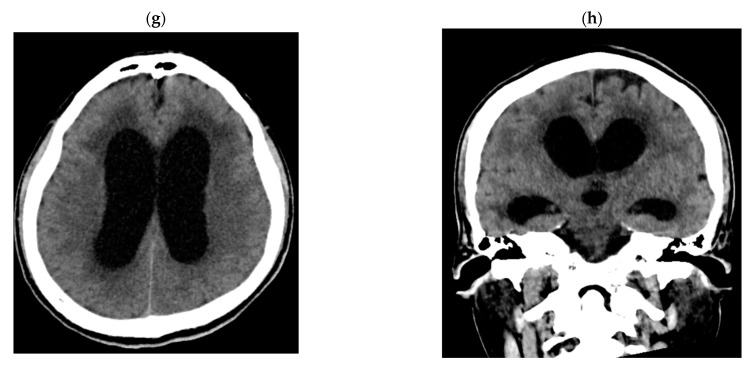
Images of case 1: (**a**) FLAIR and (**b**) T1 with gadolinium show nodular hyperintensities in the basal ganglia, with irregular annular enhancement. Case 5 images: T1-weighted images (**c**) show irregular leptomeningeal enhancement with (**d**) underlying parenchymal edema observed on FLAIR. Case 7 images: (**e**,**f**) Cerebral tomography showing subacute left parietal and occipital infarction, characterized by cortical and subcortical hypodensity. Case 8 images: (**g**,**h**) Tomography showing acute hydrocephalus with transependymal edema.

**Figure 2:**
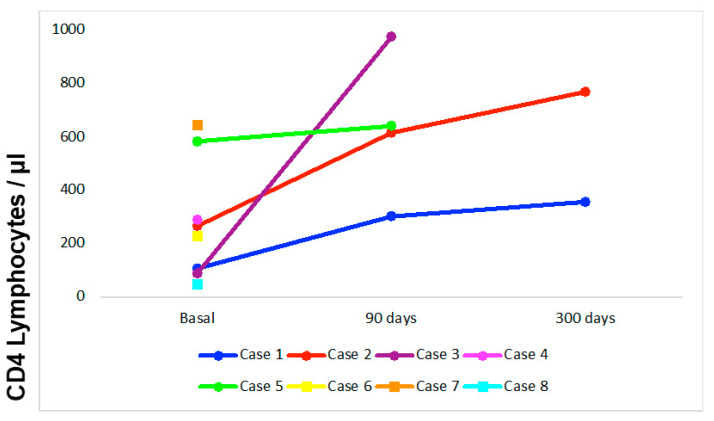
Kinetics of CD4 lymphocyte count from the time of diagnosis of cerebral cryptococcosis, up to 300 days of follow-up.

Case 1: 24-year-old female patient without comorbidities, without vaccination against COVID-19. She has a history of mild COVID-19 60 days before admission. She was admitted with a 30-day illness characterized by headache, nausea, vomiting, dysarthria, and confusion, with an 11-point Glasgow Coma Scale (GCS), nuchal stiffness, and 4/5 left hemiparesis. The CSF showed predominantly mononuclear pleocytosis, glucose 43 mg/dL (reference range: 40–76), protein: 54 mg/dL (reference range: 15–45), India ink positive, Cryptococcus latex antigen positive (1:1024), and isolation of *Cryptococcus neoformans* in Cerebrospinal Fluid (CSF) at 02 days of incubation. The blood CD4+ T lymphocyte count was 107/mm^3^ and CD8+ T lymphocytes were 253/mm^3^. Brain Magnetic Resonance Imaging (MRI) revealed cryptococomas and pseudocysts in the basal ganglia and subcortical white matter with basal leptomeningitis (Figure 1a,b). She received treatment with amphotericin B deoxycholate and fluconazole, presenting good evolution and discontinuation of therapy after five months of treatment. At three months, the CD4+ T lymphocyte count was 303/mm^3^ and CD8+ T lymphocytes were 1145/mm^3^ (Figure 2). Finally, a genomic sequencing study revealed “naked lymphocyte syndrome” during follow-up.

Case 2: 57-year-old female patient without comorbidities, without vaccination against COVID-19. She has a history of mild COVID-19 30 days before admission. She was admitted with a 17-day illness characterized by headache, nausea, vomiting, low-grade fever, confusion, and visual hallucinations. On examination, she presented a GCS of 13 points, no neck stiffness, and no focalization. The CSF showed predominantly mononuclear pleocytosis, glucose: 62 mg/dL, proteins: 39 mg/dL, negative India ink, positive Cryptococcus latex antigen (1:128), and isolation of *Cryptococcus neoformans* in CSF at 05 days of incubation. The CD4+ T lymphocyte count was 267/mm^3^ and CD8+ T lymphocytes were 143/mm^3^. Brain MRI was normal. She received treatment with amphotericin B deoxycholate and fluconazole, with good evolution and discontinuation of therapy after eight months of treatment. At three months, the CD4+ T lymphocyte count was 616/mm^3^ and CD8+ T lymphocytes were 613/mm^3^ (Figure 2).

Case 3: 58-year-old male patient, controlled diabetic, without vaccination against COVID-19. He has a history of mild COVID-19 05 months before admission. He was admitted with a disease period of 04 months characterized by headache, photophobia, nausea, vomiting, and confusion. On examination, he presents with a 14-point GCS, no nuchal stiffness, and no focusing. The CSF showed predominantly mononuclear pleocytosis, glucose: 32 mg/dL, proteins: 155 mg/dL, negative latex antigen for Cryptococcus, and isolation of *Cryptococcus neoformans* in CSF at 03 days of incubation. The CD4+ T lymphocyte count was 290/mm^3^ and CD8+ T lymphocytes were 161/mm^3^. Brain MRI was described as normal. He received treatment with amphotericin B deoxycholate and fluconazole, with good evolution and discontinuation of therapy after five months of treatment.

Case 4: 68-year-old male patient without comorbidities, without vaccination against COVID-19. He was admitted with a 14-day sick time characterized by headache, dizziness, diarrhea, confusion, on examination, mild quadriparesis, and no nuchal rigidity. He denies respiratory symptoms before admission but presents IgM and IgG (+) against SARS-CoV-2 and as RT-PCR SARS-CoV-2 positive in a nasopharyngeal swab. The CSF showed predominantly mononuclear pleocytosis, glucose: 21 mg/dL, proteins: 155, and isolation of *Cryptococcus neoformans* in CSF at 08 days of incubation. The CD4+ T lymphocyte count was 89/mm^3^ and CD8+ T lymphocytes were 20/mm^3^. Brain MRI was described as normal. He received treatment with amphotericin B deoxycholate and fluconazole, with good evolution and discontinuation of therapy after three months of treatment. At three months, the CD4+ T lymphocyte count was 976/mm^3^ and CD8+ T lymphocytes were 943/mm^3^ (Figure 2).

Case 5: 23-year-old male patient without comorbidities, without vaccination against COVID-19. He has a history of mild COVID-19 3 months before admission. He was admitted with a 22-day illness characterized by headache, nausea, vomiting, decreased visual acuity, and confusion. On examination, he presented a 14-point GCS, nuchal stiffness, bilateral VI ophthalmopareses, left homonymous hemianopsia, and 4/5 quadriparesis. The CSF showed predominantly mononuclear pleocytosis, glucose: 36 mg/dL, proteins: 35 mg/dL, India ink positive, Cryptococcus latex antigen positive (1:1024), and isolation of *Cryptococcus neoformans* in CSF at 04 days. The CD4+ T lymphocyte count was 584/mm^3^, and CD8+ T lymphocytes were 284/mm^3^, and the brain magnetic resonance evidenced symmetrical enlargement of the fissures and cisterns, with signs of basal arachnoiditis (Figure 1c,d). The patient developed obstructive hydrocephalus that required a ventriculoperitoneal shunt. He received treatment with amphotericin B deoxycholate and fluconazole, with good evolution and discontinuation of therapy after three months of treatment. At three months, the CD4+ T lymphocyte count was 642/µL and CD8+ T lymphocytes were 422/µL (Figure 2).

Case 6: 57-year-old female patient, without comorbidities, without vaccination against COVID-19. She has a history of mild COVID-19 40 days before admission. She is admitted with a 30-day sick time characterized by headache, nausea, vomiting, confusion, and visual hallucinations. On examination, she presented an 11-point GCS, nuchal rigidity, and quadriparesis 4/5. The CSF showed predominantly polymorphonuclear pleocytosis, glucose: 4 mg/dL, positive India ink, positive Cryptococcus latex antigen, and isolation of *Cryptococcus neoformans* in CSF at 05 days of incubation. The CD4+ T lymphocyte count was 228/µL and CD8+ T lymphocytes were 218/µL. Brain magnetic resonance showed subacute ischemic foci involving the body of the caudate nucleus, a posterior arm of the internal capsule, and the right cerebral peduncle. He received treatment with amphotericin B deoxycholate and fluconazole, which evolved unfavorably, presenting bacteremia associated with a central catheter due to *Klebsiella pneumoniae*, dying four weeks after admission.

Case 7: 66-year-old male patient, hypertensive, without vaccination against COVID-19. He was admitted with a 7-day sick time characterized by headache, confusion, visual hallucinations, and diaphoresis. On examination, he presented a GCS of 09 points, and right hemiplegia, without neck stiffness. He denies a history of COVID-19, but upon admission, he presents IgM and IgG (+) against SARS-CoV-2. The CSF showed predominantly mononuclear pleocytosis, glucose: 83 mg/dL, protein: 41 mg/dL, Chinese ink negative, Cryptococcus latex antigen negative, and isolation of *Cryptococcus neoformans* in CSF at 05 days of incubation. The CD4+ T lymphocyte count was 644/µL and CD8+ T lymphocytes were 186/µL. Brain tomography revealed a left parieto-occipital temporal ischemic infarction (Figure 1e,f). He received treatment with amphotericin B deoxycholate and fluconazole, presented unfavorable evolution, and presented with respiratory failure and multiple organ failure, dying seven days after admission.

Case 8: 51-year-old male patient, diabetic, without vaccination against COVID-19. He has a history of mild COVID-19 1 year before admission. He was admitted with a disease time of 335 days, characterized by unsteady gait, lateralization, headache, nausea, vomiting, progressive cognitive decline, and tonic-clonic seizures. On examination, he presented a GCS of 13 points, palsy of the right VI cranial nerve, and no nuchal rigidity. The CSF showed normal cellularity, glucose: 20 mg/dL, proteins: 64 mg/dL, positive Cryptococcus latex antigen (1:1200), and isolation of *Cryptococcus neoformans* in CSF at 02 days of incubation. The CD4+ T lymphocyte count was 46/µL, CD8+ T lymphocytes were 24/µL, and the brain tomography showed hydrocephalus with transependymal edema (Figure 1g,h). He received treatment with amphotericin B deoxycholate and fluconazole, presented unfavorable evolution with increased hydrocephalus, requiring external ventricular shunt, and later hospital-acquired pneumonia, dying eight weeks after admission. 

A detailed description of the individual cases is shown in Table 2.

## 3. Discussion

There is not yet a clear association between COVID-19 and cryptococcosis. One study determined that the incidence of cryptococcosis after hospitalization for COVID-19 was 0.022%, and in the Latino population, 0.029% [10]. However, its incidence in patients with COVID-19 who do not require hospitalization is unknown. In this series of cases, most patients had a history of mild COVID-19, were managed on an outpatient basis, and did not receive immunosuppressants.

It has been observed that the majority of patients with cryptococcosis after COVID-19 were male, were of advanced age (>60 years), and had a higher prevalence of comorbidities such as HIV infection, diabetes mellitus, and chronic kidney disease [10,11]. Likewise, it has been described that most of these patients received corticosteroids or tocilizumab [9]. In our series of cases, we observed that most patients were male, and two had a history of diabetes, but none reported having received immunosuppressive treatment before admission.

Various studies have reported that COVID-19 can cause lymphocytopenia and reduced CD4+ and CD8+ T lymphocytes (without affecting the CD4:CD8 ratio), mainly in those with severe symptoms [5,6,7]. This lymphocytopenia is secondary to the migration of peripheral T lymphocytes to organs infected by SARS-CoV-2, mainly the lungs [6]. In addition to T-cell redistribution and sequestration, it has been postulated that T-lymphocytes undergo overactivation and depletion caused by SARS-CoV-2 antigens, ultimately leading to apoptosis and pyroptosis of these T-lymphocytes [6].

Hosts with impaired T lymphocytes are more vulnerable to cryptococcosis. In the event of Cryptococcus infection, CD4+/CD8+ T lymphocytes produce IFN-γ that promotes the fungicidal activity of macrophages, which is a necessary response to control the disease. In this case series, we hypothesized that this CD4+ T lymphocytopenia-associated SARS-CoV-2 infection could increase the risk of cerebral cryptococcosis [12]. Likewise, these values were normalized in patients who obtained control of CD4+/CD8+ T lymphocytes after discharge, suggesting the transient effect produced by SARS-CoV-2 infection (Figure 2).

Contrary to what was reported by Chastain et al., who observed that most episodes of cryptococcosis occurred within ten days after the diagnosis of COVID-19, in our study, the median time was 75 days. In addition, the symptoms presented and the mortality rate seem similar to cryptococcosis not associated with COVID-19 [11]. However, those patients with cryptococcosis have higher mortality than those hospitalized for COVID-19 but without cryptococcosis [10].

We must bear in mind that all the cases in this cohort and the majority of cases reported worldwide occurred in patients who were not immunized against COVID-19. Immunization against SARS-CoV-2 may have a protective role against these coinfections. Unlike other regions, Peru has many vaccines available and has one of the best vaccination coverages against COVID-19 in the Americas [13,14]. However, there is still high resistance to the COVID-19 vaccine [15], which is a constant threat.

The main limitation of our study is that the confirmed diagnosis of COVID-19 was performed by detecting serum IgM/IgG antibodies against SARS-CoV-2 and/or RT-PCR of SARS-CoV-2 in those patients with suspicion of COVID-19 according to disease time. Due to this, most of the patients in this cohort were diagnosed by serology, which is not exempt from false positives. In addition, due to the retrospective nature of this study, it was not possible to adequately control for missing data and evaluate some variables such as nutritional status or history of exposure to Cryptococcus, smoking, and alcohol consumption, among others.

## 4. Conclusions

The relevance of this series of cases lies in the rarity of the presentation of cerebral cryptococcosis after infection with SARS-CoV-2, mainly in patients without severe symptoms and without a history of having received immunosuppressive therapy such as corticosteroids or tocilizumab. Likewise, we observed that most patients had CD4+ T lymphocytopenia, which could increase the risk of cryptococcosis in patients after SARS-CoV-2 infection. Therefore, cerebral cryptococcosis should be a differential diagnosis in patients with COVID-19, regardless of its severity, who present confusion, headache, or other neurological manifestations. 

## Figures and Tables

**Table 1:** Demographic characteristics and history, clinical characteristics, and auxiliary examinations of the study population.

Variable	All Patients (n = 8)
Demographic characteristics and background	
Age, years *	57 (37.5–62)
Sex	
- Male (%)	5 (62.50)
- Female (%)	3 (37.50)
Comorbidities	3 (37.5)
- Diabetes (%)	2 (25.00)
- Arterial hypertension (%)	1 (12.50)
- No comorbidities (%)	5 (62.50)
COVID-19 diagnosis - Positive serums antibodies (%) - RT-PCR SARS-CoV-2 positive (%)	7 (87.5) 2 (25.00)
- Days since history of COVID-19 *	75 (30–180)
COVID-19 mild	8 (100.0)
Hospitalization time (days) *	39 (27.5–55.5)
Clinical characteristics on admission	
- Illness time (days) *	26 (15.5–80)
- Headache (%)	7 (87.50)
- Nausea (%)	6 (75.00)
- Vomiting (%)	6 (75.00)
- Fever (%)	1 (12.50)
- Confusion (%)	8 (100.0)
- Hallucinations (%)	4 (50.00)
- Focal motor deficit (%)	5 (62.50)
- Seizures (%)	1 (12.50)
- Heart rate per minute *	88.5 (81–90)
- Rate respiratory per minute *	18 (18–20)
- Systolic blood pressure (mmHg) *	105 (95–125)
- Diastolic blood pressure (mmHg) *	60 (60–80)
- Mean arterial pressure (mmHg) *	75 (71.66–95)
- Temperature (°C) *	36.55 (36–36.65)
- Oxygen saturation (%) *	97 (95.5–98)
- Glasgow coma scale *	14 (13–14)
Laboratory characteristics	
- Leucocytes (cells/mm^3^) *	8935 (6830–14,000)
- Lymphocytes (cells/mm^3^) *	1230 (815–1,770)
- Platelets (cells/mm^3^) *	254,000 (209,000–295,000)
- Hemoglobin (g/dL) *	13 (12.4–14.4)
- Glucose (mg/dL) *	98 (86–107)
- Creatinine (mg/dL) *	0.48 (0.43–1.06)
- ALT (U/L) *	38 (29–74)
- AST (U/L) *	29.5 (23–38)
- CD4 lymphocyte count (cells/mm^3^) * - CD8 lymphocyte count (cells/mm^3^) * - Ratio CD4/CD8 *	247.5 (98–437) 173.5 (83.5–235.5) 1.89 (1.42–2.75)
- ELISA VIH 4th generation positive (%)	0 (0.00)
- ELISA HTLV positive (%)	0 (0.00)
- Interstitial infiltrates in chest tomography (%)	3 (37.50)

* Median and interquartile range; RT-PCR: real-time polymerase chain reaction; SARS-CoV-2: Severe Acute Respiratory Syndrome Coronavirus 2; COVID-19: Coronavirus disease 2019; TOG: aspartate aminotransferase; GPT: alanine aminotransferase, ELISA: Enzyme-Linked ImmunoSorbent Assay; HIV: human immunodeficiency virus; HTLV: human T-lymphotropic virus.

**Table 2:** Characteristics of patients with cerebral cryptococcosis after SARS-CoV-2 infection.

Case	Age	Sex	COVID Severity	Time from COVID	Illness Time	Hea-dache	Sei-zures	Alutina-tions	Cerebrospinal Fluid (CSF)						Ag. Latex (+)	Ly CD4	Ly CD8	Dead
									Cel	Mon	Prot	Gluc	Chinese Ink (+)	Culture (+)				
1	24	F	Mild	60	30	Yes	Not	Not	64	100	54	43	Yes	Yes	1024	107	253	Not
2	57	F	Mild	30	17	Yes	Not	Yes	489	100	39	62	Not	Yes	128	267	143	Not
3	58	M	Mild	180	120	Yes	Not	Not	132	100	155	32	Not	Yes	-	290	161	Not
4	68	M	Mild	-	14	Yes	Not	Yes	191	94	298	21	Not	Yes	-	89	20	Not
5	23	M	Mild	90	22	Yes	Not	Not	20	100	35	36	Yes	Yes	1024	584	284	Not
6	57	F	Mild	40	30	Yes	Not	Yes	7	0	-	4	Yes	Yes	16	228	218	Yes
7	66	M	Mild	-	7	Yes	Not	Yes	20	100	41	83	Not	Yes	-	644	186	Yes
8	51	M	Mild	365	365	Not	Yes	Not	3	100	64	20	Not	Yes	1024	46	24	Yes

CSF: cerebrospinal fluid, Cel: cellularity in CSF, Mon: percentage of mononuclear cells in CSF; prot: proteins in CSF (mg/dL), gluc: glucose in CSF (mg/dL), Ly: lymphocytes (cel/mL); M: male; F: female.

## Data Availability

The data analyzed in this manuscript, as well as its definitions, can be downloaded at the DOI: 10.17632/f9g742pkff.1.
